# Sepsis-induced coagulopathy is associated with new episodes of atrial fibrillation in patients admitted to critical care in sinus rhythm

**DOI:** 10.3389/fmed.2023.1230854

**Published:** 2023-09-15

**Authors:** Sandra Ortega-Martorell, Ivan Olier, Brian W. Johnston, Ingeborg D. Welters

**Affiliations:** ^1^School of Computer Science and Mathematics, Liverpool John Moores University, Liverpool, United Kingdom; ^2^Liverpool Centre for Cardiovascular Science, Liverpool, United Kingdom; ^3^Institute of Life Course and Medical Sciences, University of Liverpool, Liverpool, United Kingdom; ^4^Liverpool University Hospitals NHS Foundation Trust, Liverpool, United Kingdom

**Keywords:** atrial fibrillation, sepsis, machine learning, coagulopathy, database analysis, Sepsis-induced coagulopathy

## Abstract

**Background:**

Sepsis is a life-threatening disease commonly complicated by activation of coagulation and immune pathways. Sepsis-induced coagulopathy (*SIC*) is associated with micro- and macrothrombosis, but its relation to other cardiovascular complications remains less clear. In this study we explored associations between *SIC* and the occurrence of atrial fibrillation (AF) in patients admitted to the Intensive Care Unit (ICU) in sinus rhythm. We also aimed to identify predictive factors for the development of AF in patients with and without *SIC*.

**Methods:**

Data were extracted from the publicly available AmsterdamUMCdb database. Patients with sepsis and documented sinus rhythm on admission to ICU were included. Patients were stratified into those who fulfilled the criteria for *SIC* and those who did not. Following univariate analysis, logistic regression models were developed to describe the association between routinely documented demographics and blood results and the development of at least one episode of AF. Machine learning methods (gradient boosting machines and random forest) were applied to define the predictive importance of factors contributing to the development of AF.

**Results:**

Age was the strongest predictor for the development of AF in patients with and without *SIC*. Routine coagulation tests activated Partial Thromboplastin Time (aPTT) and International Normalized Ratio (INR) and C-reactive protein (CRP) as a marker of inflammation were also associated with AF occurrence in *SIC*-positive and *SIC*-negative patients. Cardiorespiratory parameters (oxygen requirements and heart rate) showed predictive potential.

**Conclusion:**

Higher INR, elevated CRP, increased heart rate and more severe respiratory failure are risk factors for occurrence of AF in critical illness, suggesting an association between cardiac, respiratory and immune and coagulation pathways. However, age was the most dominant factor to predict the first episodes of AF in patients admitted in sinus rhythm with and without *SIC*.

## 1. Introduction

Sepsis is a potentially life-threatening condition which is complicated by organ dysfunction due to a dysregulated host response to infection (1). During sepsis, invading pathogens are recognized by the innate immune system leading to the release of proinflammatory mediators into the circulation. This in turn triggers endothelial activation and recruitment of immune cells to infection sites. The interaction of innate immune cells, in particular neutrophils and monocytes with extrinsic and contact-dependent coagulation pathways, forms a major part of the body’s response to infection (2). This orchestrated activation of immune, endothelial and coagulation systems, oftendescribed as immunothrombosis, leads to macro- and microthrombotic complications and together with mitochondrial damage, energy depletion andtissue hypoxia results in cardiovascular dysfunction, including, altered myocardial contractility and arrhythmias (3). Atrial Fibrillation (AF) is the most common arrhythmia in critically ill patients with an incidence of 10–15% (4–6) in the general critical care population. In septic shock, the incidence of AF increases to over 40% (4). During sepsis, but also in patients suffering from AF, hypercoagulability is commonly observed. It is therefore warranted to investigate if the occurrence of AF in patients with sepsis is linked to an abnormal coagulation profile.

In sepsis, increased tissue factor expression, down-regulation of natural anticoagulant pathways, and hypofibrinolysis result in increased thrombin generation and clot formation in the macro- and microvasculature. Microvascular thrombosis contributes to tissue hypoxia and organ dysfunction. A recent review reported that Disseminated intravascular coagulation (DIC) is observed in 30–60% of critically ill patients with sepsis (7), depending on the score used for diagnosis. DIC is characterized by the activation of procoagulant and fibrinolytic pathways and results in intravascular micro- and macrothrombosis, which is associated with the consumption of platelets and coagulation factors (7). In later stages, bleeding is a common complication. Specifically for DIC observed in sepsis, the Sepsis-induced Coagulopathy (*SIC*) score (8) has been developed based on routine coagulation tests including platelet count and prothrombin time ratio together with components of the Sequential Organ Failure Assessment (SOFA) score (9).

In the general population, AF is associated with a prothrombotic state (10). The earlier stages of *SIC* are also characterized by hypercoagulability, which, however, differs in origin and pathophysiology. Key mechanisms of *SIC* include release of tissue factor into the circulation by activated monocytes and endothelial cells, impaired fibrinolysis, amplification of procoagulant pathways by cytokines and suppression of anticoagulant pathways. Together with the complement system, platelets trigger the formation of neutrophil extracellular traps (NETs) by activated neutrophils (11). NETs are structures consisting of histones, nuclear DNA and granule proteins secreted by neutrophils. NETs are highly prothrombotic and contribute to the procoagulant state of sepsis.

In critical illness, it remains unclear if the prothrombotic state observed in *SIC* predisposes patients to episodes of AF. Both sepsis and AF are associated with thrombotic complications including microvascular thrombosis, venous thromboembolic disease and stroke. Formation of microthrombi in small vessels is regarded as a key factor for multiorgan failure in sepsis. The pathophysiological mechanisms that link arrythmias and immunothrombosis have not been fully elucidated.

Given that coagulopathy and arrythmias are commonly observed during sepsis, we hypothesized that the incidence of AF episodes increases in patients with *SIC* compared to those without. Based on *SIC* scores we stratified patients in a large European intensive care database to investigate this hypothesis. We also aimed to describe factors associated with and predictive of the occurrence of AF episodes in septic patients admitted to ICU in sinus rhythm, discriminating between patients with *SIC* and those without.

## 2. Materials and methods

### 2.1. Data extraction

Data was extracted from the AmsterdamUMCdb, the first freely accessible European intensive care database and endorsed by the European Society of Intensive Care Medicine (ESICM) (12). Extracted variables included demographic data (e.g., age, gender, weight and height groups), vital signs (e.g., heart rate, respiratory rate, temperature, systolic blood pressure, and oxygen saturation), blood results and variables reflecting respiratory support (e.g., ventilation status, O2 concentration, Positive Endexspiratory Pressure).

Missing values were treated as described before (13), i.e., excluding admissions with more than 35% missing data, and imputing the missing data with the median (numeric variables) or the mode (categorical variables). Variables with dynamic features were converted into tabular representations by extracting their means.

The *SIC* (8), SOFA (9) and the Acute Physiology And Chronic Health Evaluation II (APACHE II) (14) scores were calculated based on data points for admission to ICU after extracting the respective variables from the database.

### 2.2. Diagnosis of sepsis

As information regarding the diagnosis of sepsis is not readily available in the AmsterdamUMCdb, we used a previously described method based on the Sepsis-3 definition (15). This enabled us to include patients with a diagnosis of sepsis before admission to ICU. We included patients in whom a diagnosis of sepsis had been coded for a time window reaching from 3 days before ICU admission until day 1 (first 24 h) of ICU admission. This time window was chosen because sepsis-induced coagulopathy is often present before ICU admission and in some cases may resolve early in the ICU stay after treatment of the underlying cause of sepsis (16).

### 2.3. Diagnosis of Sepsis-induced coagulopathy

The *SIC* score was calculated using ICU admission data points to classify patients as *SIC*-positive (*SIC* score ≥ 4) and *SIC*-negative (*SIC* score 0–3) as described previously (8). Patients with incomplete information for the calculation of the *SIC* score were excluded from this analysis.

### 2.4. Identification of AF patients

The AmsterdamUMCdb allows the identification of the heart rhythm by hourly recordings. We discriminated AF versus sinus rhythm using this variable. We only included septic patients admitted to ICU in sinus rhythm who later in their ICU stay developed an episode of AF. Variables were extracted until 1 h before the first recorded AF episode for AF patients, whereas for non-AF patients, data were analyzed for the whole ICU stay. We deliberately chose a cut-off of 1 h before the onset of AF to factor in a time window which would be required in clinical practice to conduct preventative measures (e.g., electrolyte supplementation, fluid administration). Different models were built to describe associations of physiological variables with AF episodes and to determine the importance of factors predictive of the occurrence of AF in patients admitted to ICU in sinus rhythm. We developed separate models for the following patient cohorts: all septic patients regardless of *SIC* status, patients with a diagnosis of *SIC* (*SIC*-positive) and patients without a diagnosis of *SIC* (*SIC*-negative).

### 2.5. Disease severity scores

We classified patients according to their APACHE II score into three groups: <25, 25–34, >34. An APACHE II score of 25 represents a predicted hospital mortality of 50% and a score of over 35 represents a predicted hospital mortality of 80%. SOFA classification was chosen to match the mortality estimates described before (17, 18) with scores of 0–6 representing <10% risk, 7–9 representing 15–20% risk, 10–12 representing 40–50%, 13–14 representing 50–60%, and 15 or over representing >80% risk of dying during the ICU stay.

### 2.6. Univariate and multivariate analysis

Medians and interquartile ranges were calculated for continuous variables, and frequencies and proportions were used for categorical variables. Differences between *SIC*-positive vs. *SIC*-negative septic patients were assessed using Kruskal-Wallis rank sum and Chi-square tests.

Multiple logistic regression (LR) was used to describe associations, displayed as odds ratios (OR), between physiological variables and the occurrence of AF (18). Relevant input variables were automatically selected using a sequential forward search algorithm with 3-fold cross-validation.

### 2.7. Machine learning analysis

The Machine learning (ML) algorithms random forest (RF) and gradient boosting machines (GBM) were used for prediction modeling. Both, RF and GBM are ensemble learning techniques that make their predictions by aggregating the outputs from multiple individual trees. RF repeatedly fits induction trees to many subsets of random samples with replacements extracted from the training set. In classification tasks, RF predicts a new outcome by taking the majority vote (19). Many decision trees are also used by GBM to make predictions, but unlike RF, it implements an iterative learning algorithm such that a new tree model is fitted using the instances where a previous tree performed inadequately (20). Both the RF and the GBM algorithms used the Gini impurity index to determine the ideal split at each node of their decision trees. Since they show how frequently variables were used for the splits, aggregated Gini index values offer a variable importance score (i.e., how relevant they are to the model predictions), which provides a relative rank of relevance/importance of the input variables (19).

Several RF and GBM hyperparameters were tuned using the same validation splits as for LR. For RF models, we tested a range of several variables randomly sampled between 5 and 30 and a range of minimum node size (which controls the depth of the trees) between 3 and 18. For GBM models, a range of shrinkage values (which controls the impact of each additional fitted tree) from 10^−3^ to 10^1^ and a range of minimum number of observations in a node from 3 to 18 was tested. The RF and GBM variable importance of the models with the best hyperparameter set for each cross-validation cycle was also estimated.

### 2.8. Model performance

Nested cross-validation was implemented, with the inner iterations to evaluate the variable selection, and the outer iterations to evaluate the training with the selected set of variables. Model performances were measured using the area under the receiver operator characteristic (AUC) curve. We report AUC means and confidence intervals (CI) separately for the overall cohort of septic patients, and for patients with and without *SIC*.

## 3. Results

**Figure 1:**
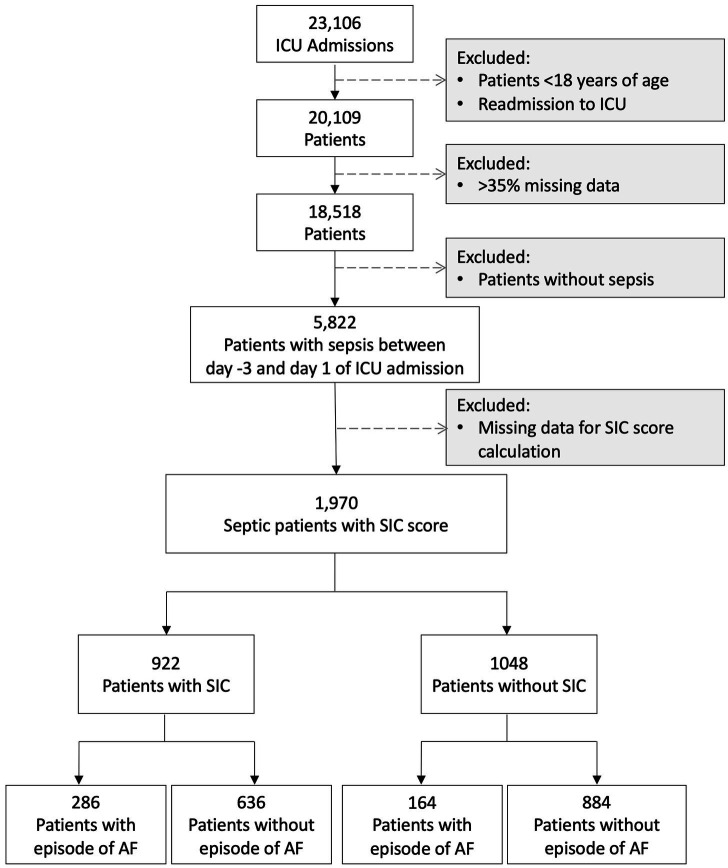
Flowchart describing the cohorts included and excluded from the analysis. ICU, intensive care unit; AF, atrial fibrillation, *SIC*, Sepsis-induced coagulopathy.

From a total of 23,106 admissions, patients <18 years of age, multiple admissions and cases with >35% missing data were excluded, resulting in 18,518 analyzable cases, of which 5,822 had sepsis recorded within day −3 to day 1 of admission (Figure 1). Of these septic patients, information to calculate the *SIC* score was available for 1,970 patients, with 922 patients fulfilling *SIC* criteria and 1,048 patients without a diagnosis of *SIC* (Figure 1). Of the 922 *SIC*-positive patients, 286 had an episode of AF, while only 164 of 1,048 patients without *SIC* developed this complication. A total of 286 septic patients with AF and 636 non-AF septic patients were diagnosed with *SIC* (*SIC* score of 4 or more), while 164 patients with AF and 884 non-AF patients were not diagnosed with *SIC*.

### 3.1. Univariate comparison between *SIC* positive and *SIC* negative patients

**Figure 2:**
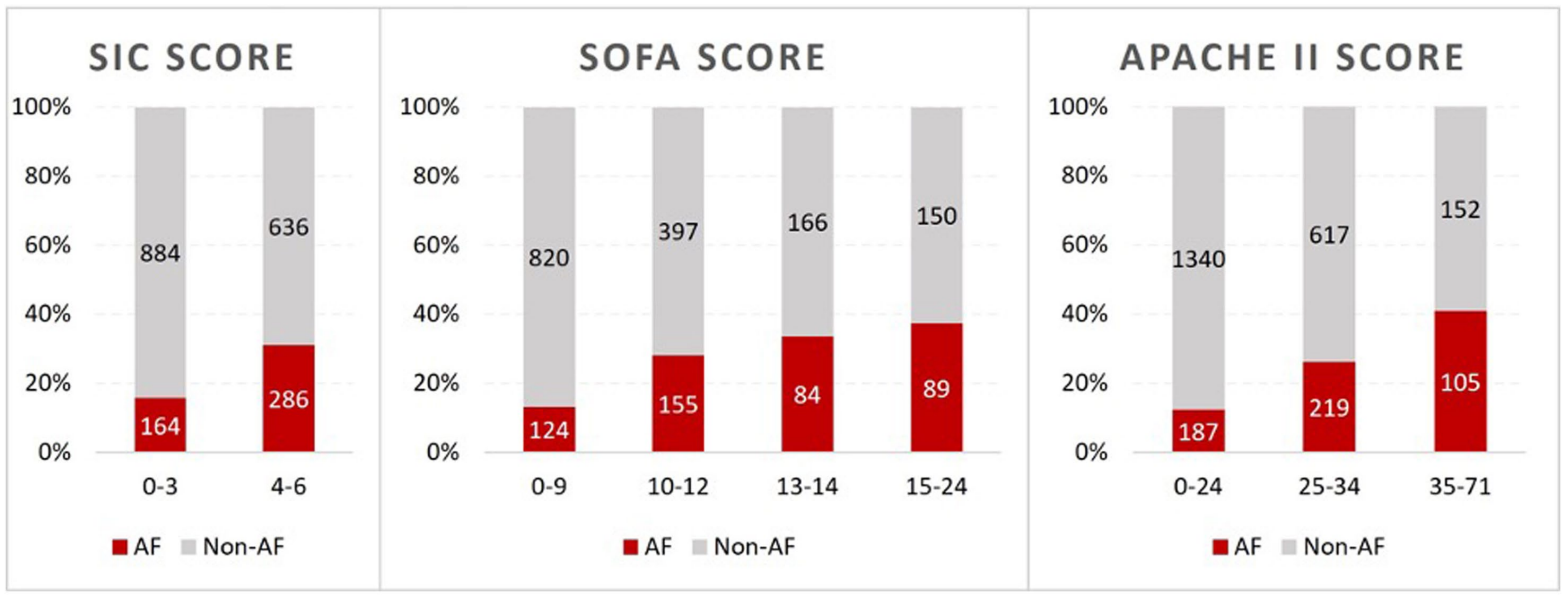
*SIC*, SOFA and APACHE II scores of AF and non-AF septic patients grouped in ranges of values. AF patients are represented in red and non-AF patients in grey. The top row shows the scores in counts and the bottom row shows them as a proportion (percentage) of AF and non-AF cases within each range of value. The bottom row shows how the proportion of AF cases increases as each of these severity scores increases.

ICU mortality was more than twice as high in *SIC*-positive patients compared to *SIC*-negative patients (28.5% versus 12.2%, *p* < 0.001). ICU length of stay was longer in *SIC*-positive patients, however, this result did not reach statistical significance. We found statistically significant differences in occurrence of AF, blood results, vital signs and blood gas analysis results between *SIC*-positive and *SIC*-negative patients (Table 1). *SIC*-positive patients developed significantly more frequent episodes of AF (286/922 patients, 31%) than patients without *SIC* (164/1048 patients, 15.6%, *p* < 0.001). Figure 2 shows the percentage of AF and non-AF patients depending on disease severity as determined by *SIC*, SOFA and APACHE II. The occurrence of AF increased with higher disease severity.

**Table 1:** Demographics, vital signs, lab results, etc. used for modeling, and routine prognostic scores.

	*SIC* positive (*N* = 922)	*SIC* negative (*N* = 1,048)	*p*-value
**Outcome**			
Length of stay [hrs]			0.060
Median	95.00	90.00	
Q1, Q3	42.00, 278.75	40.00, 215.00	
ICU mortality [yes]	263 (28.5%)	128 (12.2%)	< 0.001
AF	286 (31.0%)	164 (15.6%)	< 0.001
**Admission type**			
Location			0.351
IMC	54 (5.9%)	71 (6.8%)	
ICU and IMC	98 (10.7%)	128 (12.3%)	
ICU	760 (83.3%)	838 (80.8%)	
Urgency [unplanned]	571 (61.9%)	711 (67.8%)	0.006
**Demographics**			
Male gender	365 (39.6%)	404 (38.5%)	0.637
Age group [years]			< 0.001
18–39	141 (15.3%)	200 (19.1%)	
40–49	117 (12.7%)	130 (12.4%)	
50–59	147 (15.9%)	187 (17.8%)	
60–69	199 (21.6%)	262 (25.0%)	
70–79	206 (22.3%)	180 (17.2%)	
80+	112 (12.1%)	89 (8.5%)	
Weight group [kg]			0.914
<59	94 (10.5%)	115 (11.3%)	
60–69	176 (19.7%)	180 (17.6%)	
70–79	246 (27.5%)	276 (27.1%)	
80–89	197 (22.1%)	230 (22.5%)	
90–99	105 (11.8%)	131 (12.8%)	
100–109	42 (4.7%)	52 (5.1%)	
110+	33 (3.7%)	36 (3.5%)	
Height group [cm]			0.487
<159	43 (4.9%)	43 (4.4%)	
160–169	235 (27.0%)	253 (25.7%)	
170–179	316 (36.3%)	343 (34.9%)	
180–189	232 (26.7%)	278 (28.3%)	
190+	44 (5.1%)	66 (6.7%)	
Vital signs			
Heart rate [bpm]			< 0.001
Median	92.850	84.941	
Q1, Q3	81.139, 102.906	75.560, 95.791	
Systolic BP [mmHg]			< 0.001
Median	119.786	128.162	
Q1, Q3	109.327, 133.565	116.153, 141.512	
Temperature [°C]			< 0.001
Median	36.788	36.861	
Q1, Q3	36.270, 37.020	36.564, 37.084	
O2 saturation [%]			< 0.001
Median	80.684	86.107	
Q1, Q3	67.643, 92.324	74.958, 93.551	
ST segment [mm]			0.042
Median	0.183	0.193	
Q1, Q3	0.103, 0.293	0.120, 0.309	
Urine output [ml]			< 0.001
Median	109.447	136.759	
Q1, Q3	52.941, 161.023	99.357, 176.621	
**Arterial blood gas analysis**			
pO2 [mmHg]			0.639
Median	98.669	97.500	
Q1, Q3	85.124, 117.982	85.885, 116.043	
Arterial pH			< 0.001
Median	7.373	7.397	
Q1, Q3	7.309, 7.414	7.355, 7.429	
Anion gap [mmol/l]			< 0.001
Median	9.518	9.103	
Q1, Q3	7.500, 12.634	7.097, 10.830	
Ionised Ca++ [mmol/l]			< 0.001
Median	1.130	1.157	
Q1, Q3	1.082, 1.172	1.116, 1.193	
**Laboratory analysis**			
APTT [s]			< 0.001
Median	45.362	37.388	
Q1, Q3	39.667, 54.500	34.000, 42.490	
INR			< 0.001
Median	1.498	1.167	
Q1, Q3	1.330, 1.842	1.092, 1.246	
Hb [mmol/l]			< 0.001
Median	6.272	6.968	
Q1, Q3	5.725, 7.051	6.121, 7.833	
Leukocyte count [10^9^/l]			0.189
Median	12.500	12.700	
Q1, Q3	8.633, 17.050	9.839, 16.051	
Thrombocyte count [10^9^/l]			< 0.001
Median	153.925	235.833	
Q1, Q3	95.412, 239.952	186.267, 313.875	
ALAT [u/l]			< 0.001
Median	46.167	34.075	
Q1, Q3	23.500, 122.056	20.014, 60.750	
Creatinine [mol/l]			< 0.001
Median	101.663	74.667	
Q1, Q3	70.878, 168.688	57.694, 96.000	
CK [u/l]			0.183
Median	239.125	224.917	
Q1, Q3	94.000, 797.875	92.625, 669.656	
Blood glucose [mmol/l]			0.335
Median	7.680	7.728	
Q1, Q3	6.822, 8.673	6.984, 8.661	
CRP [mg/l]			< 0.001
Median	99.633	72.583	
Q1, Q3	41.969, 187.750	26.667, 149.500	
Calcium [mmol/l]			< 0.001
Median	1.996	2.083	
Q1, Q3	1.869, 2.112	1.972, 2.187	
Magnesium [mmol/l]			0.919
Median	0.810	0.810	
Q1, Q3	0.733, 0.903	0.747, 0.885	
Phosphate [mmol/l]			< 0.001
Median	1.117	1.005	
Q1, Q3	0.918, 1.440	0.866, 1.194	
Potassium [mmol/l]			< 0.001
Median	4.075	3.985	
Q1, Q3	3.856, 4.357	3.841, 4.173	
**Respiratory function**			
PEEP [cmH_2_O]			< 0.001
Median	8.000	7.161	
Q1, Q3	5.953, 10.535	5.099, 9.132	
O2 concentration [%]			< 0.001
Median	43.330	40.989	
Q1, Q3	39.923, 50.618	37.130, 45.905	
O2 [l/min]			0.005
Median	4.766	4.500	
Q1, Q3	3.235, 6.750	3.000, 5.967	
Insp. min. Vol. [l/min]			< 0.001
Median	9.512	8.919	
Q1, Q3	8.082, 11.104	7.650, 10.630	
Respiratory rate [breathes/min]			0.956
Median	19.502	19.402	
Q1, Q3	15.654, 23.528	16.298, 22.938	
Ventilated [yes]	760 (82.4%)	902 (86.1%)	0.026
**Scores**			
SOFA			< 0.001
0–9	295 (32.0%)	637 (60.8%)	
10–12	255 (27.7%)	296 (28.2%)	
13–14	160 (17.4%)	89 (8.5%)	
15–24	212 (23.0%)	26 (2.5%)	
APACHE II			< 0.001
0–24	386 (41.9%)	598 (57.1%)	
25–34	322 (34.9%)	344 (32.8%)	
35–71	165 (17.9%)	64 (6.1%)	

For numeric variables, median, and 25th and 75th percentiles (1st and 3rd quartile: Q1, Q3) are presented, while for categorical variables absolute numbers and percentages (%) are presented. ALAT, alanine transaminase; APTT, activated partial thromboplastin time; CK, creatine kinase; CRP, C-reactive protein; Hb, haemoglobin; IMC, intensive medium care; ICU and IMC, ICU first, then IMC; INR, international normalized ratio; PEEP, positive end-expiratory pressure.

We also observed significant differences in blood results for *SIC*-negative versus *SIC*-positive patients (Table 1). Median ALAT serum concentrations were higher in *SIC*-positive than in *SIC*-negative patients. Kidney function, measured as higher creatinine serum concentrations, was significantly worse in *SIC*-positive patients compared to *SIC*-negative patients. The cardiovascular impact of *SIC* is evidenced by significantly higher average heart rates in *SIC*-positive versus *SIC*-negative patients. In parallel, *SIC*-positive patients displayed an average systolic blood pressure that was significantly lower than in *SIC*-negative patients.

### 3.2. Multivariate logistic regression

**Figure 3:**
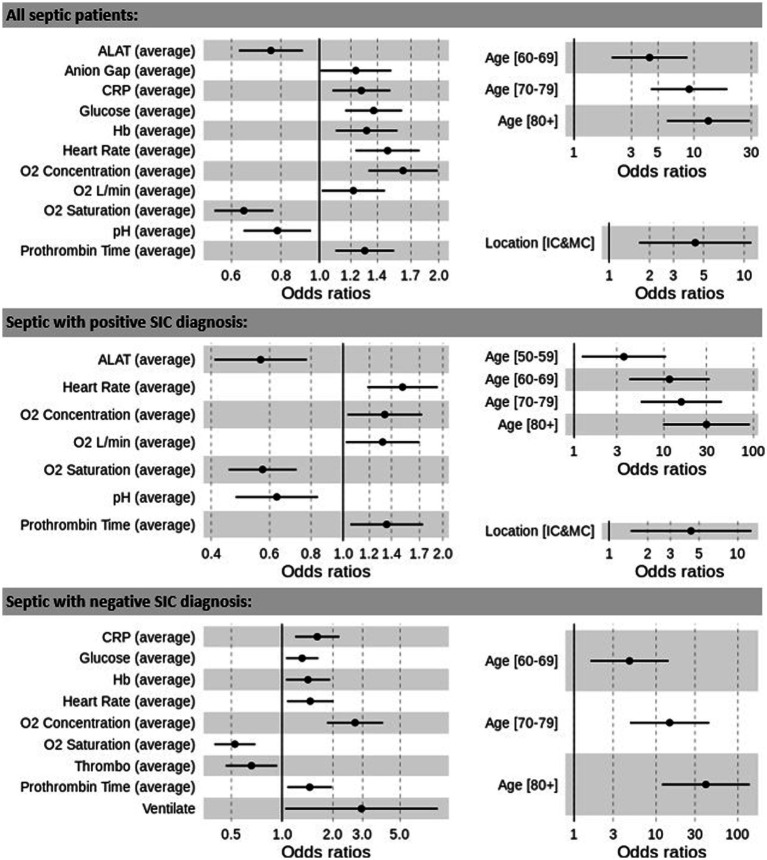
Odds ratios between significant results and prediction of AF for the overall cohort of septic patients, *SIC*-positive and *SIC*-negative patients. ALAT, alanine transaminase; CRP, C-reactive protein; Hb, haemoglobin; Ventilate, ventilated. Prothrombin time reflects international normalized prothrombin time ratio (INR).

The odds ratios (OR) for the occurence of AF in all septic patients, *SIC* positive and *SIC* negative patients are presented in Figure 3. We identified input variables (factors) that are either positively or negatively associated with the risk of AF. The most significant factor is age, with patients over 80 years having the greatest risk of developing AF (age group 80+ OR: all = 13.168, *SIC*-positive = 29.935, *SIC*-negative = 40.656).

Other factors that are positively associated with the development of AF in the three models (all septic patients, *SIC*-positive and *SIC*-negative) include heart rate (OR: all = 1.486, *SIC*-positive = 1.511, *SIC*-negative = 1.469), and INR (OR: all = 1.301, *SIC*-positive = 1.353, *SIC*-negative = 1.458). Increased oxygen requirements (O2 concentration, OR: all = 1.625, *SIC*-positive = 1.336, *SIC*-negative = 2.708, and O2 L/min, OR: all = 1.218, *SIC*-positive = 1.316), as well as reduced oxygen saturations (O2 saturation, OR: all = 0.645, *SIC*-positive = 0.572, *SIC*-negative = 0.526), were also associated with the development of AF.

Reduced levels of alanine transaminase (ALAT, OR: all = 0.755, *SIC*-positive = 0.564) and arterial pH (OR: all = 0.783, *SIC*-positive = 0.631) were associated with AF episodes in the overall sepsis and the *SIC*-positive cohorts, whilst reduced values of thrombocyte count (OR: *SIC*-negative = 0.659) increased the AF risk in the *SIC*-negative cohort. Increased values of CRP (OR: all = 1.276, *SIC*-negative = 1.616), blood glucose (OR: all = 1.370, *SIC*-negative = 1.315) and Hb (OR: all = 1.316, *SIC*-negative = 1.425) are positively associated with the development of AF in the overall sepsis and the *SIC*-negative cohorts. Anion gap was also found to be a risk factor in the overall cohort (OR: all = 1.236).

### 3.3. Variable importance analysis

**Figure 4:**
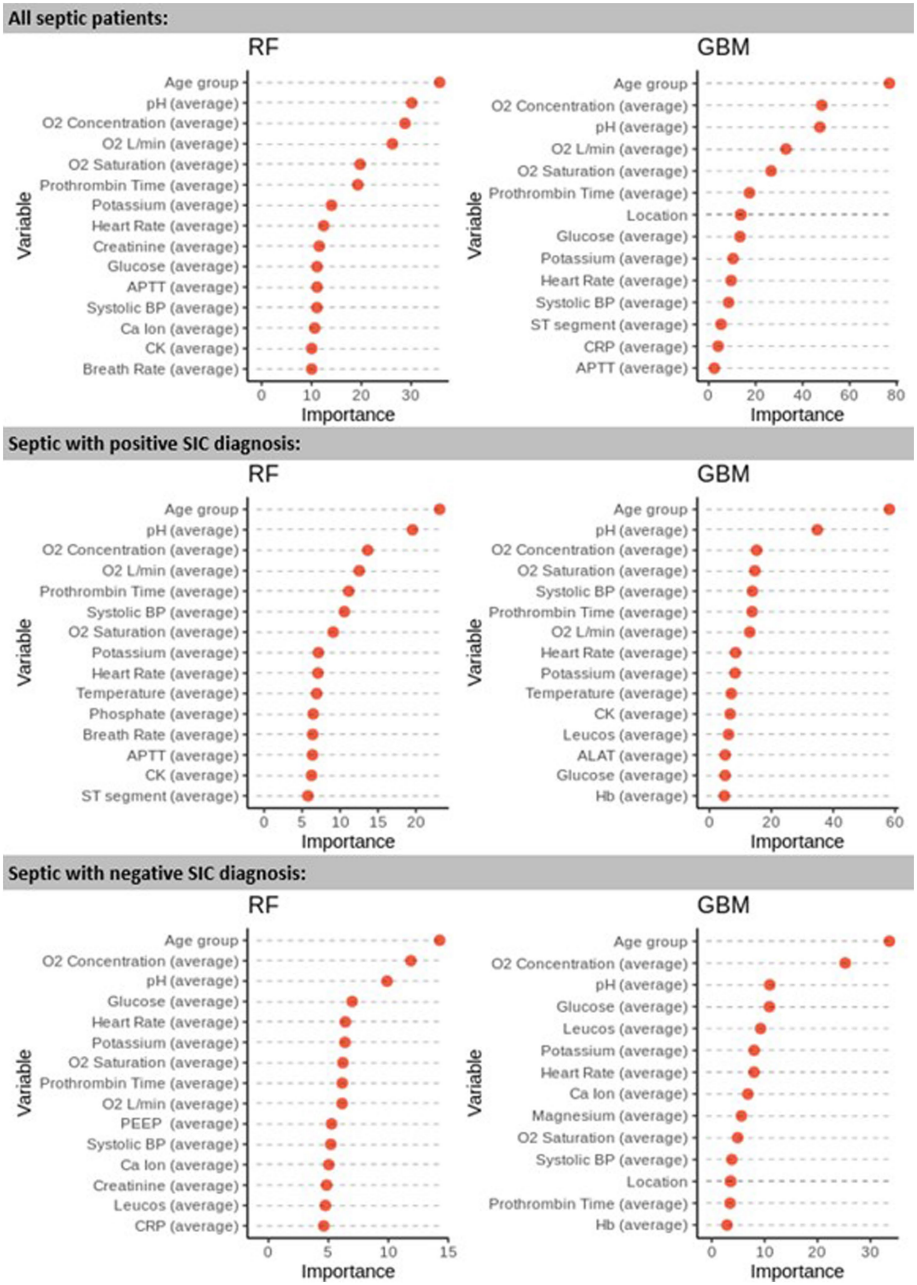
Gini variable importance as calculated by the RF and GBM models for the 3 cohorts studied: all septic patients, septic patients with *SIC* diagnosis, and non-*SIC* septic patients. Prothrombin time reflects international normalized prothrombin time ratio (INR). Abbreviations as in Table 1.

We used RF and GBM to analyse the variable importance in each cohort (Figure 4). The variation in Gini variable importance between all septic patients, septic patients with a positive *SIC* diagnosis, and septic patients with a negative *SIC* diagnosis is shown in Figure 4. Although a group of variables (e.g., age, O2 concentration, and O2 saturation) were found to be important across all models, overall, the variable rankings were different. Also, LR and the non-linear models RF and GBM identified different predictive factors depending on the patient cohort investigated (Figure 4).

### 3.4. Performance of ML and multivariate statistical models

In the three cohorts investigated (overall cohort of septic patients, *SIC*-positive and *SIC*-negative patients) LR, RF, and GBM models all demonstrated excellent performance (AUC >0.8) for prediction of the first occurrence of AF in septic patients admitted to critical care in sinus rhythm. GBM and RF outperformed LR in both *SIC* strata, but had comparable AUC characteristics for the overall cohort of septic patients (Table 2).

**Table 2:** Model performance comparisons as measured using the area under the ROC curve (AUC) for the different models developed.

	LR	RF	GBM
All septic patients	0.827 (0.795–0.859)	0.873 (0.853–0.893)	0.865 (0.848–0.882)
Septic with positive *SIC* diagnosis	0.803 (0.789–0.817)	0.859 (0.814–0.904)	0.840 (0.810–0.870)
Septic with negative *SIC* diagnosis	0.842 (0.815–0.870)	0.841 (0.807–0.875)	0.833 (0.809–0.857)

Confidence intervals are included in brackets. LR, logistic regression; RF, random forest; GBM, gradient boosting machines; SIC, Sepsis-induced coagulation.

## 4. Discussion

We demonstrate significant differences in vital signs, respiratory support, blood results and organ function in patients with *SIC* compared to those without. Our results confirm previous reports that patients who develop *SIC* when critically ill, have higher ICU mortality and longer ICU stay compared to those without *SIC*. In the subgroup analysis stratified by *SIC* diagnosis, the occurrence of AF was also associated with worse outcomes, suggesting an interaction between a first episode of AF in critically ill patients and *SIC* on mortality for patients with *SIC*.

A main finding of our work is that patients with *SIC* who are admitted to ICU in sinus rhythm, develop episodes of AF more often than those without coagulopathy. We used a *SIC* score of ≥4 to stratify septic patients into those with a diagnosis of *SIC* and those without. Several reports have demonstrated that in the general population the occurrence of AF is associated with platelet and coagulation activation (21, 22) and hypofibrinolysis (23, 24). Experimental data suggest that hypercoagulability in itself with enhanced thrombin activity as the main feature, can promote the onset of AF in mice (25). Fibrotic atrial changes and altered atrial function especially in elderly patients are established risk factors for AF in the general population but also in critically ill patients. It is therefore tempting to speculate that similar mechanisms contribute to AF observed in acutely and critically ill patients. Long et al. defined an “early coagulation disorder” within 24 h after ICU admission as a risk factor for AF in individuals with sepsis (26).

Increased fibrin formation, reduced fibrinolytic activity, platelet activation and consumption characterize coagulopathy in sepsis (27, 28). Similar changes are also observed with increasing age (29) but to a lower extent. Fibrinogen serum concentrations increase by 10 mg/dL per each decade even in healthy subjects and may contribute to the higher cardiovascular risk observed in elderly people. As acute phase reactant elevated fibrinogen levels also reflect a proinflammatory state. In sepsis high fibrinogen levels can be observed, however, the degree to which they contribute to proinflammation and hypercoagulability as dynamic processes, which are time and disease burden specific, is not fully understood. Nevertheless, during sepsis as the most severe state of acute inflammation, the cross-talk between endothelium, coagulation cascades and the immune system is now regarded as an essential process of the body’s defence (27). The association of ageing with chronic inflammation together with the increased risk of AF indicates that the cardiac-endothelial-immune axis is already activated subclinically in older individuals. When faced with severe infection, older patients are more likely to develop cardiac complications compared to younger patients due to the structural cardiovascular changes associated with ageing (30). Episodes of AF in critical illness can therefore be interpreted as the cardiac manifestation of an acute-on-chronic condition, while the occurrence of *SIC* is dominated by the severity of sepsis and the acute activation of immune and coagulation systems. This concept is supported by our observation that age is the main risk factor for AF episodes in all models developed (13).

Long et al. categorized APTT, INR and platelet count into three different severity classes to construct a 6-point score for “early coagulation disorder” and identified the presence of early coagulation disorder as a risk factor for the occurrence of AF in septic patients (26). In line with this report, our work highlights the association of septic coagulopathy with AF. Long et al. used a novel score which is not widely used in clinical practice to define “early coagulation disorder.” This limits transferability into clinical practice. Our work demonstrates that a diagnosis of *SIC* predisposes septic patients to the development of arrhythmias such as AF. In addition to demonstrating the association between coagulopathy and AF we provide comprehensive models based on machine learning and logistic regression to predict the onset of AF. We expect that such models will be integrated into modern monitoring devices and electronic patient records in the future.

Even subclinical changes in coagulation likely increase the risk of AF in septic patients. This suggests that more specific markers of coagulation may be required to better understand the role of endothelial, immune cell and coagulation cascades in the pathogenesis of AF during acute illness. However, differentiated coagulation profiles come at a cost and are currently not available as part of the routine blood panels recorded in large databases. Measurement of the coagulation disturbances in acute sepsis is complex, time-sensitive and requires serial measurements (16). aPTT and PT as measures of coagulation disregard the contribution of platelets in thrombin generation, clot formation and (immuno)thrombosis. Whole blood viscoelastic testing is becoming more widely available and further studies should include global clotting tests such as rotational thromboelastometry to fully understand the role of clinical and subclinical alterations in coagulation for the development of AF during sepsis.

The pathomechanisms how *SIC* may trigger episodes of AF remain unclear. Several of the pathways involved in the development of *SIC* have also been associated with the occurrence of arrhythmias, and in particular AF. Recently NETosis, the formation of Neutrophil Extracellular Traps (NETs), and histone release have been shown to be major contributors to thrombosis in bacterial infection (11, 31). A study linking histone levels and arrhythmias found that circulating histone levels in these patients were significantly higher in patients with new onset arrhythmias than in those without (3). Patients who developed paroxysmal AF had particularly high histone levels (3). These findings make it tempting to speculate that histones and NETs can directly affect the cardiac conduction system to trigger arrhythmias. Our finding that increased heart rates are a predictor of AF episodes could also be explained by direct effects of histones on the cardiac conduction system. Further research is required to specify such cross talk between the immune system, the coagulation cascades and the heart in more detail.

Both pathologies, sepsis and coagulopathy occur as a consequence of complex and multi-layered activation of immune, adrenergic, epigenetic, inflammatory, anti-inflammatory, endothelial and other pathways. Although we could clearly demonstrate an association between the occurrence of atrial fibrillation and sepsis-induced coagulopathy, a causal relationship between both conditions remains speculative and further research is required to explore this hypothesis.

In this study, we used conventional multivariate analysis to identify possible associations between *SIC* and a first episode of AF in patients admitted to ICU with sepsis. For many years, LR has been successfully used for predictive modeling in healthcare, showing to be a competitive algorithm when compared to other ML algorithms (32). However, as a linear model, LR has limitations when presented with non-linear data or variables with complex relationships. ML algorithms such as RF and GBM are usually better at modeling complex relationships between variables and can make more accurate predictions (33–35), contributing significantly to the development of our understanding of AF (36). ML algorithms also tend to be more robust to outliers and can handle larger datasets better. In our dataset, the ML-based models improved the performance of the LR models in *SIC*-positive and *SIC*-negative patients as evidenced by higher AUCs.

Our study has several limitations. Major limitations lie in the data set and the data that are recorded in the database. Data about a previous history of AF are not available in the AmsterdamUMC database, hence it was not possible to discriminate between new onset and pre-existing AF. However, since the recurrence of AF after its first occurrence in critical illness is frequently observed (37) it is likely that acute triggers unmask underlying chronic structural changes and a chronically altered cardiovascular function. Therefore any episode of AF may be regarded as the clinical manifestation of a chronic underlying pathological process. The coagulation results available in the AmsterdamUMCdb are limited to routinely performed tests such as aPTT, platelet count and prothrombin time ratios. Hence more specific markers of *SIC*, including D-Dimer and fibrinogen, which are likely to improve the performance of our models further, are not available in a sufficient number of patients to allow statistical analysis. Changes in aPTT, platelet count and INR are complex and vary depending and underlying cause of infection, premorbid condition and treatment. Impaired synthetic capacity, but also consumption of coagulation factors, Vitamin K deficiency or anticoagulants may prolong aPTT or increase INR. The lack of data for specific coagulation tests in the Amsterdam database make a more detailed analysis of the underlying subtype of coagulopathy impossible. Lastly, the presence of *SIC* may reflect mainly the severity of disease instead of representing a separate distinct risk factor for the development of AF. Investigating different non-septic patient cohorts with disseminated intravascular coagulation regarding their risk profile for AF may help to clarify the role of the endothelial-immune-coagulation axis for the development of AF in acute illness.

For patients that were invasively ventilated, O2 concentration was documented, while O2 in L/min was recorded for patients on non-invasive ventilatory support. We refrained from converting Oxygen supply in l/min into FiO2 due to the lack of universally accepted conversion formulas.

We have not yet externally validated the models developed which limits the generalisability and transferability to other settings. Further research is also required to evaluate how the implementation of such models can support clinical decision-making at the bedside.

## 5. Conclusion

Our results confirm previous studies that the occurrence of AF in patients during critical illness and *SIC* is associated with higher mortality. Higher INR, increased heart rate and more severe respiratory failure were also identified as risk factors, suggesting an association between cardiac, respiratory and coagulation systems. Despite the influence of routine coagulation markers and cardiorespiratory parameters, age was the most dominant factor to predict the first episodes of AF in patients admitted in sinus rhythm with and without *SIC*.

## Data availability statement

Publicly available datasets were analyzed in this study. This data can be found at: https://amsterdammedicaldatascience.nl/amsterdamumcdb/.

## Ethics statement

Ethical approval was not required for the study involving humans in accordance with the local legislation and institutional requirements. Written informed consent to participate in this study was not required from the participants or the participants’ legal guardians/next of kin in accordance with the national legislation and the institutional requirements.

## Author contributions

SO-M extracted and prepared the data. SO-M and IO conducted the statistical and machine learning analysis, and evaluated the results. IW and BJ provided the clinical expertise. IW and SOM conceptualised the study. All authors were involved in the study design and the selection of relevant variables from the dataset and contributed to the writing, reviewing and editing, and approved the final manuscript.
